# Red and white blood cell morphology characterization and hands-on time analysis by the digital cell imaging analyzer DI-60

**DOI:** 10.1371/journal.pone.0267638

**Published:** 2022-04-27

**Authors:** Oh Joo Kweon, Yong Kwan Lim, Mi-Kyung Lee, Hye Ryoun Kim

**Affiliations:** Department of Laboratory Medicine, Chung-Ang University College of Medicine, Seoul, Republic of Korea; The Ohio State University, UNITED STATES

## Abstract

**Background:**

The Sysmex DI-60 digital morphology analyzer is a fully automated, cell-locating image analysis system. This study aimed to evaluate the analytical performance of DI-60.

**Methods:**

A total of 822 peripheral blood smears were used. The diagnostic performance of DI-60 in terms of red blood cell (RBC) morphology characterization, white blood cell (WBC) differentials, and the total assay time including hands-on time was evaluated.

**Results:**

In comparison with manual slide review, DI-60 demonstrated acceptable accuracy in recognizing polychromasia, target cells, and ovalocytes. However, for schistocytes, DI-60 demonstrated low specificity (10.4%) despite the high sensitivity (97.2%). In the precision analysis of RBC morphology characterization, borderline samples harboring specific RBCs showed inconsistencies in the positive results among 20 replicates. Particularly, 6 of 10 samples showed inconsistencies in the precision for schistocytes. For WBC differentials, the overall agreement between pre-classification results and user-verified results was 89.4%. Except for basophils, normal WBCs showed a good correlation between DI-60 (after user verification) and manual counts. The sensitivities in detecting immature granulocytes, blasts, atypical lymphocytes, and normoblasts were 85.9%, 92.0%, 37.5%, and 77.6%, respectively. Although the total assay time of DI-60 was longer than that of manual review, the hands-on time was considerably shorter with a difference of 144.1 s/slide for abnormal samples.

**Conclusion:**

DI-60 demonstrated acceptable performance for normal samples. However, for abnormal WBC differentials and RBC morphology characterization, it should be utilized carefully. DI-60 may contribute to an improvement in laboratory efficiency with increased feasibility.

## Introduction

Complete blood counts (CBCs), white blood cell (WBC) differentials, and red blood cell (RBC) morphology characterization using peripheral blood smears (PBSs) are important and essential components of the clinical management of patients. Although automated hematology analyzers have replaced microscopes for the majority of differential counts of normal WBCs in most laboratories, a manual differential count on a Romanowsky-stained PBS remains the gold standard for differential counts [1]. Moreover, most laboratories still heavily depend on manual differentials especially for abnormal samples because automated counters do not provide differential counts specifically for abnormal WBCs [2]. RBC morphology characterization via PBSs is also essential in diagnostic hematology, especially for the evaluation of the cause of anemia and follow-up evaluation during treatment [1]. However, RBC morphology characterization has not been possible with conventional hematology analyzers, which only provide limited information about abnormal morphology in the form of “flags” [3].

Nevertheless, manual PBS review also has several drawbacks, such as time-consuming and labor-intensive procedures, requirements for a skilled technician, and substantial reviewer subjectivity [4, 5].

In recent years, automated instruments for the morphological assessment of PBSs have become a powerful modality that allows for the uniform, fast, and robust characterization and quantification of peripheral blood cells [6]. These instruments can be especially useful in laboratories facing labor shortages and may be considered for economic feasibility. The Sysmex DI-60 digital morphology analyzer (Sysmex, Kobe, Japan) automatically locates cells on a PBS, takes an image of each identified cell, and performs WBC differential counts along with RBC morphology characterization [4, 7].

Although there have been studies related to the DI-60 performance in WBC differentials [4, 8], those focusing on the precision of RBC morphology characterization and/or the hands-on time are relatively limited. In this study, we evaluated the DI-60 performance with a focus on the accuracy of RBC morphology characterization, RBC morphology precision, and the hands-on time, as well as the accuracy of WBC differential counts compared with manual differential counts.

## Materials and methods

### Study samples, CBC analysis, and blood film preparation

This study was conducted in Chung-Ang University Hospital, Seoul, Republic of Korea, from Dec 2019 to Aug 2020. Among the patient blood samples for routine CBC analysis, 822 randomly selected samples (531 for WBC analysis and 291 for RBC analysis) were evaluated. Among the 531 samples for WBC analysis, 34 samples were derived from healthy individuals who showed unremarkable findings on routine health check-up including CBC profiles. In addition, among the 291 samples for RBC analysis, 32 samples were derived from normal individuals.

All blood samples were collected in K_2_ EDTA anticoagulant tubes (Becton, Dickinson and Company, Franklin Lakes, NJ). CBC analysis was performed with Sysmex XN-20 modules (Sysmex), and blood smear slides were prepared by the fully automated slide preparation unit. Sysmex SP-10 (Sysmex). The staining protocol was as follows: 2 min for Wright pure time, 3 min for Wright dilute (1:10) time, 5 min for Giemsa dilute (1:25) time, 15 s for rinse time, and 10 min for dry time.

### DI-60

DI-60 is an automated digital cell morphology system that uses artificial neural network technology to locate, identify, and pre-classify WBCs and pre-characterize RBCs. It comprises an automated microscope that scans the PBS, a digital camera that captures the images of all cellular and particulate material on the slide, and a computer that classifies each image using a complex algorithm. DI-60 can be connected directly to the analyzer track of the Sysmex CBC analyzer along with SP-10. The software version of the evaluated DI-60 instrument was 6.0.4.

DI-60 can be used for the analysis of RBC morphological characteristics (target cells, schistocytes, helmet cells, sickle cells, spherocytes, elliptocytes, ovalocytes, teardrop cells, stomatocytes, acanthocytes, ecchinocytes, Howell-Jolly bodies, pappenheimer bodies, and basophilic stippling) and report polychromasia, hypochromasia, anisocytosis, microcytosis, macrocytosis, and poikilocytosis. The cut-off criteria for RBC morphology grading can be adjusted by users. For example, if the cut-off of “ovalocytes 3+” and “ovalocytes 2+” is set as “>20%” and “11–20%”, respectively, samples harboring 15% of ovalocytes can be classified as “ovalocytes 2+”. If the cut-off of “ovalocytes 3+” is adjusted or lowered to”10%”, the samples can be classified as “ovalocytes 3+”. In addition, the RBC morphology characterized by DI-60 can be re-characterized cell by cell by users.

DI-60 classifies WBCs as band neutrophils, segmented neutrophils, lymphocytes, monocytes, eosinophils, basophils, promyelocytes, myelocytes, metamyelocytes, blasts, variant lymphocytes (or atypical lymphocytes), and plasma cells and non-WBCs as normoblasts, giant platelets, platelet aggregates, smudge cells, and artifacts. “Unidentified” is a class of cells and objects that the system cannot identify [4]. The results of pre-classification must be verified or re-classified and confirmed by medical technologists; otherwise, DI-60 was programmed not to report the results. The number of WBCs counted can be set as 100 or 200 cells, and in this study, the 200 cells option was used for WBC differential counts.

### Study design

The clinical performance of DI-60 in RBC analysis, WBC analysis, and hands-on time analysis was investigated. For manual slide review, two experienced medical technologists reviewed the blood smear using a light microscope and performed differential counts (total 200 cells/slide) in an ideal zone of the PBS according to CLSI H20-A2 [9].

For RBC analysis, performance evaluation included (1) accuracy assessment of the RBC morphology determined by DI-60 (pre-characterization) compared with the manual PBS review following the International Council for Standardization in Hematology (ICSH) recommendations (cut-off criteria for the grading of DI-60 RBC parameters were adjusted according to the ICSH recommendations) [1] and (2) assessment of the repeatability and within-laboratory precision of percentage values generated from DI-60 (pre-classification) for the RBC morphology of PBSs from 20 representative abnormal samples according to CLSI EP5-A3 [10]. To analyze the parameters of RBCs with hypochromasia, anisocytosis, microcytosis, and macrocytosis, the mean corpuscular hemoglobin concentration (MCHC), red cell distribution width (RDW), and mean corpuscular volume (MCV) from XN-20 were compared with those from DI-60 and not the manual slide review according to the ICSH recommendations [1].

For WBC analysis, performance evaluation included (1) comparison of WBC and non-WBC (normoblasts, giant platelets, smudge cells, artifacts, and unidentified cells) differential counts between pre-classification by DI-60 and after user verification, (2) analysis of the correlation between verified DI-60 WBC differential counts and manual differential counts, (3) assessment of the accuracy of detecting abnormal cells (immature granulocytes (metamyelocytes, myelocytes, and promyelocytes), blasts, atypical lymphocytes, and normoblasts) between DI-60 and manual review, and (4) assessment of the agreement of DI-60 results to the flags generated from the hematology analyzer (XN-20) for abnormal cell detection.

Analysis of the total assay time including hands-on time was conducted using 10 abnormal samples and 10 normal samples. The total assay time was measured 5 times per slide for DI-60, and a manual review was conducted for 200 WBC differential counts by two well-trained medical technicians who did not operate the DI-60 instrument. The hands-on time for DI-60 was designated as the time interval from WBC verification to the final report of WBC differential counts. The hands-on time for manual slide review was designated as the time interval from the time of putting the PBS on the microscope to the time of the final report of 200 WBC differential counts. In other words, the hands-on time is the same as the total assay time in manual slide review.

### Statistical analysis

The Kolmogorov-Smirnov test was performed to test the normality of the data distribution. To investigate the correlation between nonparametric values from DI-60 and manual slide review, Spearman’s rank test was performed by calculating Spearman’s rho (r_s_). As all quantitative data showed a nonparametric distribution, Mann-Whitney U test was performed to investigate the similarity of the distribution between DI-60 and manual counts. For qualitative comparison, the sensitivity, specificity, positive percent agreement (PPA), negative percent agreement (NPA), total agreement (%), and Cohen’s kappa values were assessed by 2-by-2 crosstab analysis. The kappa values were interpreted according to the criteria proposed by Landis and Koch [11]. Repeatability and within-laboratory precision were assessed in 2 runs per day (2 replicates per run) for 5 days, and the coefficient of variances was calculated according to CLSI EP5-A3 [10].

Statistical analyses were performed using SPSS software (v.19; IBM, Armonk, NY) and Microsoft Excel (v. 2016; Microsoft, Redmond, WA). A *P* value of ≤0.05 was considered statistically significant.

### Ethics statement

This study was complied with all relevant national regulations, institutional policies and is in accordance with the tenets of the Helsinki Declaration (as revised in 2013), and has been approved by the Chung-Ang University Hospital Institutional Review Board (IRB, approval number 2140-003-460). All samples were fully anonymized, and the need for informed consent was waived according to the IRB policy.

## Results

### RBC analysis

A comparison of RBC morphology characterization between DI-60 and manual slide review is shown in Table 1. For polychromasia, target cells, and ovalocytes, sensitivities were higher than 87.5% with specificities higher than 86.9%. For schistocytes, although the sensitivity was 97.2%, the specificity was only 10.4%, generating 89.6% of the false-positive results. Likewise, the sensitivity for anisocytosis was 100.0%; however, the specificity was significantly low at 6.3%. In contrast, for spherocytes, elliptocytes, and teardrop cells, sensitivities were lower than 33.3% with excellent specificities (higher than 99.3%).

**Table 1:** Comparison of RBC morphology characterization between DI-60 and manual slide review with 291 peripheral blood smears.

	Agreement rate		Sensitivity, % (95% CI)	Specificity, % (95% CI)	Positive rate, % (N)	
	Total, % (95% CI)	±1 grade ^†^, % (95% CI)			DI-60	Manual or XN
Polychromasia	93.8 (90.4–96.1)	93.8 (90.4–96.1)	100.0 (38.3–100.0)	93.8 (90.3–96.1)	7.2 (21/291)	1.0 (3/291)
Hypochromia^‡^	92.8 (89.2–95.3)	93.5 (90.0–95.8)	13.6 (3.9–34.2)	100.0 (98.3–100.0)	1.0 (3/291)	7.6 (22/291)
Anisocytosis^‡^	20.6 (16.4–25.7)	27.8 (23.0–33.3)	100.0 (93.5–100.0)	6.3 (3.7–10.3)	95.2 (277/291)	23.0 (67/291)
Microcytosis^‡^	96.2 (93.3–98.0)	96.6 (93.7–98.2)	9.1 (0.0–39.9)	100.0 (98.4–100.0)	0.3 (1/291)	3.8 (11/291)
Macrocytosis^‡^	47.8 (42.1–53.5)	48.5 (42.1–54.2)	100.0 (51.1–100.0)	57.6 (41.8–53.3)	53.3 (155/291)	1.7 (5/291)
Target cells	98.3 (95.9–99.9)	98.6 (96.4–99.6)	100.0 (51.1–100.0)	98.6 (96.3–99.6)	3.1 (9/291)	1.7 (5/291)
Schistocytes	28.9 (24.0–34.3)	39.9 (34.4–45.6)	97.2 (91.8–99.4)	10.4 (6.7–15.7)	92.4 (269/291)	37.1 (108/291)
Spherocytes	99.0 (96.9–99.8)	99.0 (96.9–99.8)	33.3 (5.6–79.8)	100.0 (98.4–100.0)	0 (0/291)	1.0 (3/291)
Elliptocytes	99.3 (97.4–99.9)	99.3 (97.4–99.9)	33.3 (5.6–79.8)	100.0 (98.4–100.0)	0.3 (1/291)	1.0 (3/291)
Ovalocytes	86.6 (82.2–90.1)	86.9 (85.6–90.4)	87.5 (62.7–97.7)	86.9 (82.4–90.4)	17.2 (50/291)	5.5 (16/291)
Teardrop cells	96.6 (93.7–98.2)	96.9 (94.1–98.5)	12.5 (0.1–49.2)	99.3 (96.1–99.9)	1.0 (3/291)	2.8 (8/291)
Acanthocytes	97.3 (94.6–98.7)	97.3 (94.6–98.7)	N/A^§^	97.3 (94.6–98.7)	2.8 (8/291)	0 (0/291)

Abbreviations: CI, confidence interval

^†^Within “mod/2+” and “many/3+” (except for schistocytes, within “few/1+” ~ “many/3+”).

^‡^For the parameters of cells with hypochromia (MCHC), anisocytosis (RDW), microcytosis (MCV), and macrocytosis (MCV), data from XN-20 were compared with those from DI-60 instead of manual slide review according to ICSH guidelines.

^§^N/A, not assessed due to lack of positive samples from manual slide review.

Of the 10 samples in the assessment of the repeatability and within-laboratory precision of RBC morphology values (%) generated from DI-60, samples with borderline results indicating the presence of abnormal cells are listed in Table 2. Borderline samples were designated as samples harboring abnormal cells near the positive cut-off (1% (0.0–2.0%) for schistocytes and 5% (2.5–7.5%) for polychromatic cells, target cells, schistocytes, spherocytes, elliptocytes, ovalocytes, teardrop cells, acanthocytes, and basophilic stippling). Very major RBC morphology characteristics of borderline samples showed inconsistent results among 20 replicates (negative to positive or positive to negative results) with varying degrees of repeatability and within-laboratory precision. The detailed results of the repeatability and within-laboratory precision of RBC morphologies obtained with DI-60 for all samples are shown in S1 Table. Notably, schistocytes showed inconsistent results for 6 of 10 samples.

**Table 2:** Assessment of repeatability and within-laboratory precision of DI-60 using peripheral blood smears with borderline^†^ results for abnormal RBC morphologies among 20 replicates.

	Mean, %	Repeatability, CV, % (95% CI)	Within-laboratory precision, CV, % (95% CI)	Result consistency
Polychromatic cells	3.94	18.3 (12.8–32.2)	23.1 (16.9–36.4)	Neg~2+
	6.22	24.6 (17.2–43.2)	27.1 (20.5–40.1)	Neg~2+
	6.76	37.7 (26.3–66.1)	41.4 (31.2–61.2)	Neg~2+
Target cells	5.21	32.4 (22.6–56.8)	38.6 (29.0–57.9)	Neg~2+
Schistocytes	0.42	80.8 (56.5–141.8)	96.0 (72.0–143.9)	Neg~1+
	0.67	27.1 (18.9–47.5)	40.3 (29.2–65.0)	Neg~1+
	1.24	36.2 (25.3–63.5)	38.7 (29.2–57.2)	Neg~1+
	1.55	44.8 (31.3–78.5)	51.4 (38.9–76.1)	Neg~2+
	1.63	19.0 (13.3–33.3)	23.7 (17.6–36.0)	Neg~2+
Spherocytes	5.70	47.2 (33.0–82.8)	55.1 (40.7–85.2)	Neg~2+
Elliptocytes	2.92	11.8 (8.2–20.6)	12.9 (9.8–19.1)	Consistent
Ovalocytes	3.74	18.7 (13.1–32.9)	33.2 (23.8–54.8)	Neg~2+
	7.49	21.4 (15.0–37.6)	33.4 (24.2–53.8)	Neg~2+
Teardrop cells	2.61	15.3 (10.7–26.9)	19.7 (14.4–31.1)	Consistent
	4.14	18.1 (12.7–31.8)	19.2 (14.5–28.3)	Neg~2+
	4.39	14.5 (10.1–25.4)	17.1 (12.8–25.6)	Neg~2+
Acanthocytes	3.19	58.5 (40.9–102.7)	71.9 (53.6–109.5)	Neg~2+
	6.50	26.5 (18.5–46.5)	31.7 (23.8–47.5)	Neg~2+
Basophilic stippling	3.83	31.8 (22.2–55.8)	38.3 (28.3–59.3)	Neg~2+
	5.30	28.5 (19.9–50.0)	31.3 (23.6–46.2)	Neg~2+

Abbreviations: CV, coefficient of variation

^†^Near the cut-off value: 1% (0–2.0%) for schistocytes and 5% (2.5–7.5%) for others.

### WBC analysis

A comparison of WBC and non-WBC differential counts between pre-classification by DI-60 and after user verification is shown in Table 3. Neutrophils, lymphocytes (including lymphocyte variants or atypical lymphocytes), monocytes, eosinophils, normoblasts, giant platelets, and smudge cells showed an acceptable agreement (higher than 91.3%) between pre-classification and verification results. However, the agreement of band neutrophils was only 5.6%, and 88.1% of the cells that DI-60 pre-classified as band neutrophils were revealed to be neutrophils after their verification. Likewise, the agreement of promyelocytes and myelocytes was lower than 42.0%. The overall agreement between pre-classification results and user-verified results was 89.4%.

**Table 3:** Comparison of white blood cell (WBC) and non-WBC differential counts between pre-classification by DI-60 and after verification by users.

	Verification														
Pre-classification	Band (%)	Neu (%)	Lym (%)	Mono (%)	Eos (%)	Baso (%)	Promyel (%)	Myel (%)	Meta (%)	Blasts (%)	nRBCs (%)	Giant PLT (%)	Smudge (%)	Artifacts (%)	Kappa (95% CI)
Band (N = 7002^†^)	**5.6**	88.1	0.1	0.2	4.4	0.0	0.0	0.0	0.9	0.0	0.0	0.0	0.7	0.0	0.09 (0.07–0.11)
Neu (N = 115725)	0.2	**98.3**	0.1	0.0	0.9	0.0	0.0	0.0	0.0	0.0	0.0	0.0	0.5	0.0	0.88 (0.88–0.89)
Lym (N = 29762)	0.0	0.0	**96.1**	0.1	0.0	0.0	0.0	0.0	0.0	0.1	0.0	0.0	3.6	0.0	0.94 (0.94–0.94)
Mono (N = 9099)	0.2	1.3	0.6	**92.1**	0.1	0.0	0.0	0.0	0.1	0.0	0.0	0.0	5.4	0.0	0.86 (0.86–0.87)
Eos (N = 3621)	0.0	2.1	0.0	0.0	**97.0**	0.0	0.0	0.0	0.2	0.0	0.0	0.1	0.6	0.0	0.78 (0.77–0.79)
Baso (N = 2199)	0.2	4.8	19.8	0.4	0.5	**63.6**	0.0	0.4	0.5	0.2	0.0	0.1	9.5	0.1	0.75 (0.73–0.76)
Promyelo (N = 595)	0.2	1.8	0.2	20.7	2.2	0.3	**35.3**	11.8	16.8	1.2	0.0	0.0	9.6	0.0	0.52 (0.47–0.57)
Myelo (N = 1914)	1.2	8.9	11.9	3.7	4.2	0.7	0.1	**42.0**	23.6	0.5	0.0	0.5	2.8	0.0	0.56 (0.54–0.58)
Meta (N = 265)	1.1	14.7	9.4	1.9	1.1	0.0	0.0	0.0	**62.6**	0.0	0.0	0.4	8.7	0.0	0.24 (0.19–0.28)
Blasts (N = 3593)	0.0	0.0	13.1	10.7	0.0	0.0	0.5	1.3	2.0	**70.8**	0.0	0.0	1.5	0.1	0.81 (0.80–0.82)
nRBCs (N = 1547)	0.0	0.1	2.8	0.0	0.0	0.0	0.0	0.1	0.0	0.0	**91.3**	3.3	0.6	1.9	0.94 (0.93–0.95)
Giant PLT (N = 9981)	0.0	0.0	0.0	0.0	0.0	0.0	0.0	0.0	0.0	0.0	0.0	**99.5**	0.3	0.1	0.99 (0.99–0.99)
Smudge (N = 9375)	0.0	0.1	0.1	0.0	0.4	0.0	0.0	0.0	0.1	0.0	0.0	0.0	**99.1**	0.2	0.81 (0.80–0.81)
Artifacts (N = 3187)	0.0	1.0	1.3	0.0	0.1	0.0	0.0	0.1	2.6	0.0	0.0	0.6	26.5	**67.7**	0.79 (0.77–0.80)
UnID (N = 6352)	1.9	45.1	12.0	19.8	5.2	1.7	0.0	0.3	2.0	0.9	0.7	1.5	8.5	0.5	-

Abbreviations: Band, band neutrophils; Neu, neutrophils; Lym, lymphocytes; Mono, monocytes; Eos, eosinophils; Baso, basophils; Promyel, promyelocytes; Myel, myelocytes; Meta, metamyelocytes; nRBCs, normoblasts; Giant PLT, giant platelets; Smudge, smudge cells; UnID, unidentified

^†^Total cell count

A comparison of the differential counts between DI-60 (verified by user) and manual slide review is shown in Table 4. The median differential counts of neutrophils (including band neutrophils), basophils, immature granulocytes (metamyelocytes, myelocytes, and promyelocytes), and atypical lymphocytes were higher with DI-60 compared with manual review (*P*<0.05 for all). In contrast, the differential counts of monocytes and normoblasts were lower with DI-60 compared with manual review (*P*>0.01 for both). Spearman’s rank test revealed that the r_s_ values for neutrophils, lymphocytes, monocytes, eosinophils, and basophils were 0.96, 0.94, 0.76, 0.87, and 0.37, respectively. The r_s_ values for atypical lymphocytes, blasts, and normoblasts were 0.44, 0.63, and 0.46, respectively.

**Table 4:** Comparison of WBC and normoblast differential counts between DI-60 (after user verification) and manual slide review.

Cells	Median value, % (IQR)			Mean difference, % (95% CI)	Spearman’s rank test	
	Manual	DI-60	*P*		r_s_	*P*
Neutrophils^†^	66.5 (52.5–76.5)	69.4 (54.5–80.3)	0.01	2.76 (0.45 to 5.07)	0.96	<0.01
Lymphocytes	18.5 (11.0–30.5)	17.0 (9.6–30.4)	0.07	-1.22 (-3.21 to 0.77)	0.94	<0.01
Monocytes	6.5 (5.00–9.5)	5.2 (3.2–8.1)	<0.01	-1.51 (-2.11 to -0.91)	0.76	<0.01
Eosinophils	1.5 (0.5–3.5)	1.1 (0.0–3.9)	0.38	0.12 (-0.59 to 0.85)	0.87	<0.01
Basophils	0.0 (0.0–0.5)	0.5 (0.0–0.5)	<0.01	0.27 (0.20 to 0.34)	0.37	<0.01
Immature granulocytes^‡^	0.0 (0.0–1.0)	0.0 (0.0–1.5)	0.03	0.14 (-0.17 to 0.45)	0.76	<0.01
Blasts	0.0 (0.0–0.0)	0.0 (0.0–0.0)	0.48	-0.82 (-1.70 to 0.06)	0.63	<0.01
Atypical lymphocytes	0.0 (0.0–0.0)	0.0 (0.0–0.0)	0.04	0.10 (0.03–0.17)	0.44	<0.01
Normoblasts	0.0 (0.0–0.0)	0.0 (0.0–0.0)	<0.01	-0.57 (-0.78 to 1.94)	0.46	<0.01

Abbreviations: IQR, interquartile range; CI, confidence interval

^†^Including band neutrophils.

^‡^Including metamyelocytes, myelocytes, and promyelocytes.

The sensitivities of DI-60 in detecting immature granulocytes (metamyelocytes, myelocytes, and promyelocytes), blasts, atypical lymphocytes, and normoblasts were 85.9% (176/205, 80.4–90.0%), 92.0% (23/25, 73.9–98.9%), 37.5% (24/64, 26.7–49.8%), and 77.6% (38/49, 64.0–87.1%), respectively (values indicate the number of slides with positive results from DI-60/manual slide review, 95% CI). The specificities were 78.1% (253/324, 73.3–82.3%), 98.5% (515/523, 97.0–99.3%), 90.1% (419/465, 87.0–92.5%), and 82.5% (396/480, 78.8–85.7%), respectively. The kappa values between DI-60 and manual slide review for immature granulocytes, blasts, atypical lymphocytes, and normoblasts were 0.62 (0.55–0.68), 0.81 (0.70–0.93), 0.27 (0.15–0.38), and 0.36 (0.27–0.46), respectively. Data on the accuracy of DI-60 for detecting abnormal WBCs and normoblasts are presented in S2 Table.

The results of assessing the agreement of DI-60 results to the flags generated from XN-20 for detecting abnormal cells showed that the PPAs of DI-60 for the flags “NRBC Present”, “IG Present”, and “Left Shift?” were 82.9%, 79.5%, and 82.7%, respectively. In contrast, those for the flags “Blasts?” and “Atypical Lympho?” were 46.8% and 30.9%. Details of the results regarding the agreement between the DI-60 results and XN-20 flags are shown in S3 Table.

### Hands-on time

The results of hands-on time analysis with 10 abnormal samples and their detailed CBC profiles are shown in Table 5. When the analysis was performed consecutively (user verification was performed immediately while another analysis was in progress), the hands-on time of DI-60 was considerably shorter than that of manual slide review (780.6 s vs. 2222 s, *P*<0.05) with a difference of 1441.4 s (144.1 s/slide). However, the total assay time of DI-60 was longer than that of manual slide review (2645.8 s vs. 2422 s, *P*<0.05).

**Table 5:** Total assay time and hands-on time comparison between DI-60 and manual slide review with 10 peripheral blood smears made from abnormal blood samples.

Sample no.	WBCs		RBCs		Assay time^†^, mean s (min–max)			
	Absolute count (Neu-Lym-Mono %)	Other findings	Hb (MCV-MCHC-RDW)	nRBCs	DI-60^‡^			Manual Hands-on time^§^
					Analysis	Hands-on time	Total	
1	13260 (78-5-14-1)	IG (+)	6.0 (78.2–34.9–19.9)	+	221.6 (207–233)	84.6 (62–104)	306.2 (269–333)	197.5 (190–205)
2	34220 (86-2-9-1)	IG (+)	7.3 (76.3–29.8–25.1)	+	204.4 (197–209)	69 (57–85)	273.4 (259–294)	185 (175–195)
3	6670 (66-28-4-2)	-	6.7 (131.3–35.4–19.8)	+	207.4 (198–225)	56.8 (46–80)	264.2 (252–305)	199 (191–207)
4	4830 (75-16-5-1)	IG (+)	9.9 (89.7–38.1–18.6)	+	282.8 (261–305)	91.8 (81–98)	374.6 (342–400)	243.5 (223–264)
5	5400 (85-9-6-0)	-	9.0 (93.0–30.9–19.9)	+	237.2 (235–241)	67.0 (57–91)	303.8 (293–332)	179.5 (168–191)
6	4640 (51-37-8-4)	-	6.4 (58.4–26.0–29.2)	-	247.6 (228–259)	66.2 (54–90)	314.2 (285–350)	211 (199–223)
7	4670 (49-33-11-7)	-	7.9 (114.4–31.1–19.3)	-	252.2 (226–290)	70.6 (62–83)	322.8 (297–352)	209.5 (205–214)
8	2240 (15-68-16-0)	-	13.6 (75.5–32.2–16.5)	-	612.8 (580–662)	94.4 (70–117)	707.2 (665–748)	332 (310–354)
9	5470 (56-35-6-1)	-	11.3 (90.5–33.0–17.1)	-	340.6 (322–357)	76.6 (72–81)	417.2 (399–435)	199 (189–209)
10	17360 (69-14-12-1)	IG (+) Blasts (+)	5.7 (103.4–31.1–22.3)	-	206 (194–221)	103.6 (84–129)	309.6 (291–323)	266 (260–272)
Total (consecutive^¶^)					2812.6 (2648–3002)	780.6 (645–958)	2645.8 (2553–2747)	2222 (2110–2334)

Abbreviations: WBCs, white blood cells; RBCs, red blood cells; IG, immature granulocytes (metamyelocytes, myelocytes, and/or promyelocytes); Neu, neutrophils; Lym, lymphocytes; Mono, monocytes; Hb, hemoglobin; MCV, mean corpuscular volume; MCHC, mean corpuscular hemoglobin concentration; RDW, red cell distribution width

^†^ Time interval from the slide input to the final report of the result.

^‡^Measured five times per slide.

^§^ Including 200 manual WBC differential counts performed by two well-trained medical technicians independently.

^¶^ User verification was performed immediately while another analysis was in progress.

Similarly, for all 10 normal samples, compared with manual slide review, DI-60 showed a longer total assay time but a considerably shorter hands-on time. The total assay time of DI-60 was 2411.2 s (2245–2596 s) including 410 s (404–464 s) of hands-on time, whereas that of manual slide review was 1856 s (1803–1909 s). The difference in the hands-on time between DI-60 and manual review was 1446 s (144.6 s/slide), similar to that required for abnormal samples.

## Discussion

In this study, we evaluated the analytical performance of DI-60 with a focus on RBC morphology characterization and their precision, WBC differential counts, and the hands-on time. The precision of RBC morphology characterization and the hands-on time have rarely been investigated in the literature. This study was conducted in a tertiary hospital, which performs 800–1000 CBCs per day with manual reviews of 200–300 PBSs per day.

Evaluation of the accuracy of RBC morphology characterization showed that DI-60 was reliable in RBC morphology characterization for polychromasia, ovalocytes, and target cells (>87.5% sensitivity and >86.9% specificity). However, for teardrop cells, its sensitivity was only 12.5%, thus generating significant false-negative results. In addition, sensitivities for spherocytes and elliptocytes were as low as 33.3%; nevertheless, considering that there were a few positive samples in the study, further investigation with those cells is needed. In contrast, for schistocytes, it showed sufficiently high sensitivity but poor specificity (10.4%), which could generate false-positive results. Schistocytes are a diagnostic feature of microangiopathic hemolytic anemia disorders such as thrombotic thrombocytopenic purpura and hemolytic uremic syndrome as well as for their follow up [12]. Therefore, the ICSH recommended a more sensitive semi-quantitative grading system for schistocytes compared with other cells, with the “few/1+” grade only for schistocytes; on the other hand, other cells are graded by a two-tiered system, with “mod/2+” and “many/3+’ [1, 12]. Our results indicated the high sensitivity of DI-60 in classifying RBCs as schistocytes; thus, the DI-60 cut-off values for grading should be adjusted or increased to reduce false-positive results considering the high workload in routine analysis. In addition, in our experience, a number of artifacts may be mischaracterized as schistocytes by DI-60; thus, the algorithm of DI-60 used to classify cells as schistocytes should be further improved for accuracy. The need for adjusting grading cut-off values for schistocytes and other RBCs has also been suggested in other studies [8, 13]. Because the ICSH criteria are not mandatory guidelines, each laboratory can use different grading criteria for manual PBS review. In such circumstances, the cut-off adjustment for DI-60 is also necessary to produce the same results as obtained from manual PBS. Moreover, the algorithm of DI-60 for the characterization of abnormal RBCs can be different in view of not only the ICSH guidelines but also the individual standards of each laboratory.

The RBC parameters of cells with hypochromia, anisocytosis, microcytosis, and macrocytosis showed poor sensitivity and specificity. Nevertheless, as the ICSH recommended that the MCHC, RDW, and MCV generated from the hematology analyzer should be used in the report of these parameters [1] instead of those from manual microscopic review, the poor performance of these parameters would be irrelevant.

In the analysis of the precision of RBC morphology characterization, samples having borderline ranges of morphology characteristics showed inconsistent results for most morphologies except for elliptocytes. Following the ICSH recommendations, the results for polychromatic cells, target cells, spherocytes, ovalocytes, teardrop cells, acanthocytes, and basophilic stippling were inconsistent among 20 replicates. Based on these findings, laboratories should pay attention to the borderline results of RBC morphologies from DI-60 in their final report. Specifically, because the schistocyte grading system is divided sophisticatedly (<1% for 1+, 1–2% for 2+, and >2% for 3+), the results among replicates could vary not only for borderline samples but also for negative or positive samples; among 10 tested samples, 6 samples showed inconsistencies in the positive results, and 2 samples showed inconsistencies in the grading. This outcome could be related to the accuracy of DI-60 in recognizing schistocytes as described above; thus, the overall improvement of DI-60 for schistocyte recognition is crucial.

The overall agreement of WBC differentials between pre-classification by DI-60 and after user verification was 89.4%, and this value was similar to that in previous studies using DI-60 [4, 8] and its sister instrument, the CellaVision DM96 system (Cydac, Uppsala, Sweden) [14, 15]. Except for basophils, normal WBCs showed an acceptable agreement (higher than 92.1%) between pre-classification results and user-verified results. However, the agreement of band neutrophils was only 5.6%, and the majority (88.1%) of the cells that DI-60 classified as band neutrophils were verified as segmented neutrophils. In addition, the agreement rates of promyelocytes, myelocytes, metamyelocytes, and artifacts were lower than 70%; because users cannot adjust the algorithm of DI-60 for classifying those cells, a technician should carefully verify the DI-60 classification of these cells. Similar findings were also reported in a previous study [4].

A comparison between DI-60 (verified) WBC differential counts and manual differential counts by microscopy showed unreliable performance for basophils. DI-60 classified more cells as basophils (false-positive results); consequently, DI-60 differential counts were higher than manual counts with a low correlation coefficient (r_s_) of 0.37. Other studies also reported similarly low correlation coefficients for basophils with DM96 [14, 16]. Neutrophil and monocyte differential counts were significantly different between DI-60 and manual slide review; however, their differences and r_s_ values were considered to be within acceptable ranges. For abnormal cells, atypical lymphocytes and normoblasts had poor correlation coefficient values (0.44 and 0.46, respectively). Consequently, the sensitivity of DI-60 in detecting atypical lymphocytes and normoblasts was unsatisfactory (37.5% and 77.6%, respectively). The kappa values for these cells were also low (0.27 and 0.36, respectively), which were interpreted as “fair agreement” [11]. Based on these findings, manual differential counts are still important to report accurate differential counts, especially for basophils, atypical lymphocytes, and normoblasts.

Because automated hematology analyzers generate “flags” to indicate the potential presence of abnormal cells, we compared XN-20 flags with DI-60 results. For all of the investigated flags, kappa values between XN flags and DI-60 were lower than 0.54, generating up to 16.6% of XN Flags (-)/DI-60 (+) results. Therefore, DI-60 could be used in combination with XN flags to screen for abnormal cells; however, manual PBS review is still necessary considering the accuracy of DI-60 for abnormal cells.

In hands-on time analysis, compared with manual slide review, DI-60 required considerably less hands-on time for both abnormal and normal samples (144.1 s/slide and 144.6 s/slide for abnormal and normal samples, respectively). Therefore, DI-60 can improve the efficiency of hematology laboratories by saving time for manual slide review, which would be beneficial especially for less experienced technologists and laboratories facing labor shortages. However, unlike the hands-on time, the total assay time of DI-60 was longer with all tested PBSs. Particularly, a sample with a WBC count of 2,240/μL required 612.8 s (10 min and 12.8 s) for analysis in DI-60 (even without verification), which is considered to be unacceptably long for routine tests; this may be attributed to the low WBC count of the sample. Therefore, the time may be shortened by adjusting the number of WBCs counted by DI-60 from 200 to 100 cells. Another drawback of DI-60 was that it did not provide the coordinates of a specific cell. In other words, users cannot review the cells displayed by DI-60 through manual microscopy, and a considerable amount of training time and experience is required to verify the results with confidence. Considering these characteristics of DI-60, elaborate workflow processes including slide-making rules and criteria for DI-60 analysis and/or manual slide review should be developed for each hematology laboratory.

This study has several limitations. First, some cells such as promyelocytes, spherocytes, and elliptocytes were not analyzed in depth. In addition, there were no positive samples for sickle cells and stomatocytes. Second, we did not assess the platelet enumeration function of DI-60. Platelet count estimation by DI-60 was found to be satisfactory with a good correlation to XN data in another study [17]. Third, as we did not connect SP-10 to DI-60, the efficiency of their connection cannot be evaluated. Laboratory workflows with an automated line system that integrates the steps from blood cell counting to cell morphological analysis are expected to further improve laboratory efficiency. Finally, because the studied samples were selected randomly, it was impossible to evaluate the DI-60 performance according to patient characteristics.

In conclusion, an automated cell imaging analyzer, DI-60, demonstrated acceptable performance in RBC morphology characterization and WBC differentials for normal samples. In addition, DI-60 may contribute to the improvement of laboratory efficiency with increased feasibility. However, for abnormal samples, it should be utilized carefully with other indicators such as CBCs and flags from the hematology analyzer. Caution is also required when reporting the borderline results of RBC morphologies considering the imprecision of DI-60.

## Supporting information

S1 Table Precision analysis of RBC morphology characterization by DI-60 for 10 peripheral blood smear slides with 20 replicates (2 replicates per run, 2 runs per day, 5 days). (PDF)

S2 Table Accuracy of DI-60 compared with manual slide review for detecting the abnormal WBCs and normoblasts of 531 peripheral blood slides. (PDF)

S3 Table Assessment of the agreement of DI-60 results to the flags generated from XN-20 for detecting abnormal cells. (PDF)
