# Supplementary material for: Red and white blood cell morphology characterization and hands-on time analysis by the digital cell imaging analyzer DI-60
Source: PLoS One. 2022 Apr 27;17(4):e0267638. doi: 10.1371/journal.pone.0267638 (PMC9045635; doi:10.1371/journal.pone.0267638)
Supplement: S1 Table — (PDF) [file pone.0267638.s001.pdf]

Supplementary Table 1. Precision analysis of RBC morphology characterization by DI-60 for 10 peripheral blood smear slides with 20 replicates (2 replicates per run, 2 runs per day, 5 days)

Morphology (positive cut-off†)	Sample no.	Mean, %	Repeatability, %CV (95% CI)	Within-laboratory precision, %CV (95% CI)	Grade consistency
Polychromatic cells (5%)	1	6.76	37.7 (26.3–66.1)	41.4 (31.2–61.2)	Neg~2+
	2	2.30	15.2 (10.6–26.7)	17.2 (12.9–25.9)	Consistent
	3	6.22	24.6 (17.2–43.2)	27.1 (20.5–40.1)	Neg~2+
	4	2.00	32.2 (22.5–56.6)	40.9 (30.5–62.3)	Consistent
	5	2.88	25.2 (17.6–44.3)	30.6 (22.9–45.8)	Consistent
	6	0.28	68.3 (47.7–119.8)	74.8 (56.5–110.6)	Consistent
	7	2.74	28.7 (20.0–50.4)	33.6 (25.2–50.4)	Consistent
	8	0.73	30.4 (21.2–53.3)	36.7 (27.1–56.8)	Consistent
	9	1.47	41.5 (29.0–72.8)	49.4 (37.1–74.0)	Consistent
	10	3.94	18.3 (12.8–32.2)	23.1 (16.9–36.4)	Neg~2+
Target cells (5%)	1	0.60	36.6 (25.6–64.2)	39.2 (29.6–57.9)	Consistent
	2	12.81	19.1 (13.4–33.6)	21.8 (16.5–32.2)	Consistent
	3	1.63	39.0 (27.3–68.5)	43.4 (32.8–64.2)	Consistent
	4	24.02	11.8 (8.3–20.7)	16.1 (11.6–25.9)	2+~3+
	5	0.20	38.3 (26.7–67.2)	65.1 (46.1–110.6)	Consistent
	6	5.21	32.4 (22.6–56.8)	38.6 (29.0–57.9)	Neg~2+
	7	0.15	58.2 (40.6–102.1)	63.7 (48.1–94.2)	Consistent
	8	0.68	26.0 (18.2–45.7)	30.0 (22.2–46.4)	Consistent
	9	14.67	32.7 (22.9–57.4)	35.9 (27.1–53.1)	2+~3+
	10	0.51	39.7 (27.8–69.7)	43.0 (32.5–63.5)	Consistent
Schistocytes (1%)	1	25.59	14.4 (10.0–25.2)	15.6 (11.8–23.1)	Consistent
	2	1.55	44.8 (31.3–78.5)	51.4 (38.9–76.1)	Neg~2+
	3	3.38	34.7	37.8	Consistent

			(24.2–60.8)	(28.4–56.7)	
	4	0.42	80.8 (56.5–141.8)	96.0 (72.0–143.9)	Neg~1+
	5	1.63	19.0 (13.3–33.3)	23.7 (17.6–36.0)	Neg~2+
	6	3.69	51.7 (36.1–90.7)	62.9 (46.9–95.8)	1+~2+
	7	2.11	41.6 (29.1–73.1)	53.2 (39.6–80.9)	Neg~2+
	8	0.67	27.1 (18.9–47.5)	40.3 (29.2–65.0)	Neg~2+
	9	1.24	36.2 (25.3–63.5)	38.7 (29.2–57.2)	Neg~1+
	10	2.20	28.2 (19.7–49.6)	41.2 (30.2–65.0)	1+~2+
Spherocytes (5%)	1	5.70	47.2 (33.0–82.8)	55.1 (40.7–85.2)	Neg~2+
	2	0.07	86.8 (60.6–152.3)	96.4 (72.8–142.6)	Consistent
	3	0.00	Not Accessible	Not Accessible	Consistent
	4	0.00	Not Accessible	Not Accessible	Consistent
	5	0.00	316.2 (221.0–555.0)	447.2 (320.7–738.2)	Consistent
	6	0.02	142.5 (99.6–250.1)	180.6 (134.5–274.9)	Consistent
	7	0.00	447.2 (312.5–784.8)	447.2 (337.9–661.4)	Consistent
	8	0.01	159.7 (111.6–280.3)	268.0 (194.3–431.8)	Consistent
	9	0.02	208.9 (146.0–366.6)	244.9 (185.1–362.2)	Consistent
	10	0.24	36.8 (25.7–64.6)	54.7 (39.2–90.3)	Consistent
Elliptocytes (5%)	1	0.01	258.2 (180.4–453.1)	325.4 (242.4–495.3)	Consistent
	2	0.16	27.3 (19.1–48.0)	40.1 (29.1–64.6)	Consistent
	3	0.10	100.6 (70.3–176.5)	114.1 (85.7–171.1)	Consistent
	4	0.02	223.6 (156.3–392.5)	267.8 (201.0–401.5)	Consistent
	5	0.54	18.5 (12.9–32.5)	28.5 (20.4–47.0)	Consistent
	6	0.52	31.5 (22.0–55.3)	35.5 (26.8–52.5)	Consistent
	7	0.49	32.8 (23.0–57.6)	41.7 (30.8–64.5)	Consistent
	8	2.92	13.8 (8.2–20.6)	17.9 (9.8–19.1)	Consistent
	9	0.07	68.6 (47.9–120.3)	76.7 (57.5–115.0)	Consistent
	10	0.53	38.8 (27.1–68.1)	69.8 (46.2–142.1)	Consistent
Ovalocytes	1	0.36	40.7	54.1	Consistent

(5%)			(28.5–71.5)	(39.6–85.3)	
	2	0.75	83.2 (58.1–145.9)	107.4 (79.3–166.2)	Consistent
	3	11.73	33.6 (23.4–58.9)	37.9 (28.2–57.7)	Consistent
	4	0.51	33.6 (21.2–56.9)	55.4 (39.2–94.0)	Consistent
	5	9.85	15.1 (10.5–26.4)	17.9 (13.4–27.3)	Consistent
	6	0.93	61.8 (43.2–108.4)	74.5 (53.4–123.0)	Consistent
	7	14.92	22.1 (15.5–38.9)	25.6 (19.2–38.4)	Consistent
	8	7.49	21.4 (15.0–37.6)	33.4 (24.2–53.8)	Neg~2+
	9	2.22	41.7 (29.2–73.3)	49.1 (36.8–73.6)	Consistent
	10	3.74	18.7 (13.1–32.9)	33.2 (23.8–54.8)	Neg~2+
Teardrop cells (5%)	1	0.24	53.0 (37.1–93.1)	59.9 (44.6–91.2)	Consistent
	2	1.30	28.5 (19.9–50.0)	31.6 (23.7–47.3)	Consistent
	3	4.14	18.1 (12.7–31.8)	19.2 (14.5–28.3)	Neg~2+
	4	0.58	34.9 (24.4–61.3)	42.2 (30.9–66.6)	Consistent
	5	8.09	7.3 (5.1–12.8)	10.5 (7.7–16.5)	Consistent
	6	1.46	21.8 (15.2–38.2)	26.1 (19.6–39.2)	Consistent
	7	4.39	14.5 (10.1–25.4)	17.1 (12.8–25.6)	Neg~2+
	8	1.11	10.2 (7.1–17.9)	18.2 (12.9–30.9)	Consistent
	9	0.70	21.0 (14.7–36.8)	23.8 (17.8–36.3)	Consistent
	10	2.61	15.3 (10.7–26.9)	19.7 (14.4–31.1)	Consistent
Acanthocytes (5%)	1	6.50	26.5 (18.5–46.5)	31.7 (23.8–47.5)	Neg~2+
	2	1.21	62.4 (43.6–109.4)	72.4 (54.3–108.5)	Consistent
	3	1.29	43.2 (30.2–75.7)	46.5 (34.9–69.7)	Consistent
	4	0.60	63.5 (44.4–111.5)	93.9 (65.6–164.7)	Consistent
	5	0.16	112.0 (78.3–196.5)	124.0 (93.1–185.9)	Consistent
	6	1.04	88.8 (62.1–155.9)	109.4 (80.1–172.5)	Consistent
	7	3.19	58.5 (40.9–102.7)	71.9 (53.6–109.5)	Neg~2+
	8	1.13	43.1	61.8	Consistent

			(30.1–75.7)	(45.3–97.5)	
	9	0.28	96.9 (67.7–170.1)	118.3 (88.8–177.4)	Consistent
	10	0.46	83.0 (58.0–145.6)	103.6 (76.5–160.3)	Consistent
Basophilic stippling (5%)	1	32.98	17.4 (12.2–30.6)	19.3 (14.6–28.5)	Consistent
	2	0.67	43.5 (30.4–76.3)	66.0 (45.4–120.5)	Consistent
	3	0.63	49.9 (34.9–87.6)	58.1 (42.6–91.7)	Consistent
	4	3.83	31.8 (22.2–55.8)	38.3 (28.3–59.3)	Neg~2+
	5	0.67	31.1 (21.7–54.6)	43.3 (31.7–68.3)	Consistent
	6	0.02	149.5 (104.4–262.3)	162.3 (121.8–243.3)	Consistent
	7	0.30	39.9 (27.9–70.1)	51.8 (37.6–83.5)	Consistent
	8	0.75	32.2 (22.5–56.5)	37.3 (27.8–56.7)	Consistent
	9	5.30	28.5 (19.9–50.0)	31.3 (23.6–46.2)	Neg~2+
	10	1.79	24.8 (17.3–43.6)	30.5 (22.3–48.1)	Consistent

†Following the International Council for Standardization in Hematology recommendations (*Int. Jnl. Lab. Hem*. 2015, 37, 287–303
