# Supplementary material for: Red and white blood cell morphology characterization and hands-on time analysis by the digital cell imaging analyzer DI-60
Source: PLoS One. 2022 Apr 27;17(4):e0267638. doi: 10.1371/journal.pone.0267638 (PMC9045635; doi:10.1371/journal.pone.0267638)
Supplement: S2 Table — (PDF) [file pone.0267638.s002.pdf]

Supplementary Table 2. Accuracy of DI-60 compared with manual slide review for detecting the abnormal WBCs and normoblasts of 531 peripheral blood slides

	Sensitivity, % (95% CI)	Specificity, % (95% CI)	Total agreement rates, % (95% CI)	Kappa (95% CI)
Metamyelocytes	81.4 (75.3–86.3)	79.2 (74.5–83.2)	80.0 (76.3–83.2)	0.77 (0.73–0.81)
Myelocytes	59.4 (50.5–67.6)	92.1 (89.1–94.4)	84.5 (81.2–87.3)	0.54 (0.46–0.63)
Promyelocytes	N/A[a]	99.2 (98.0–99.8)	99.2 (98.0–99.8)	N/A
Immature granulocytes [b]	85.9 (80.4–90.0)	78.1 (73.3–82.3)	81.1 (77.5–84.2)	0.62 (0.55–0.68)
Blasts	92.0 (73.9–98.9)	98.5 (97.0–99.3)	98.2 (96.6–99.1)	0.81 (0.70–0.93)
Atypical lymphocytes	37.5 (26.7–49.8)	90.1 (87.0–92.5)	83.7 (80.4–86.7)	0.27 (0.15–0.38)
Normoblasts	77.6 (64.0–87.1)	82.5 (78.8–85.7)	82.0 (78.5–85.1)	0.36 (0.27–0.46)

Abbreviations: CI, confidence interval; N/A, not assessed

[a] Not assessed due to lack of positive samples from manual slide

[b] metamyelocytes, myelocytes, and promyelocytes
