# Supplementary material for: Red and white blood cell morphology characterization and hands-on time analysis by the digital cell imaging analyzer DI-60
Source: PLoS One. 2022 Apr 27;17(4):e0267638. doi: 10.1371/journal.pone.0267638 (PMC9045635; doi:10.1371/journal.pone.0267638)
Supplement: S3 Table — (PDF) [file pone.0267638.s003.pdf]

Supplementary Table 3. Assessment of the agreement of DI-60 results to the flags generated from XN-20 for detecting abnormal cells

XN flags	PPA	NPA	Total agreement	Kappa	XN flags (+) DI-60 (+)	XN flags (-) DI-60 (+)	XN flags (+) DI-60 (-)	XN flags (-) DI-60 (-)
"NRBC Present"	82.9 (68.4–91.8)	82.0 (78.4–85.2)	82.1 (78.6–85.1)	0.34 (0.25–0.44)	6.4% (34/531)	16.6% (88/531)	1.3% (7/531)	75.7% (402/531)
"IG Present"	79.5 (73.6–84.4)	75.6 (70.6–80.1)	77.2 (73.5–80.6)	0.54 (0.47–0.61)	32.2% (171/531)	14.5% (77/531)	8.3% (44/531)	45.0% (239/531)
"Blasts?"	46.8 (33.3–60.8)	97.9 (96.2–98.9)	93.4 (91.0–95.3)	0.52 (0.39–0.67)	4.1% (22/531)	1.9% (10/531)	4.7% (25/531)	89.3% (474/531)
"Left Shift?"	82.7 (70.0–90.9)	83.7 (80.1–86.8)	83.6 (80.2–86.5)	0.42 (0.32–0.51)	8.1% (43/531)	14.7% (78/531)	1.7% (9/531)	75.5% (401/531)
"Atypical Lympho?"	30.9 (21.1–42.7)	89.2 (86.0–91.7)	81.7 (78.2–84.8)	0.20 (0.09–0.31)	4.0% (21/531)	9.4% (50/531)	8.9% (47/531)	77.8% (413/531)

Abbreviations: PPA, positive percent agreement; NPA, negative percent agreement
